# Shoutai pills for treating recurrent miscarriage: a systematic review and meta-analysis of the safety and clinical efficacy in 11 randomized controlled trials

**DOI:** 10.3389/fphar.2025.1540073

**Published:** 2025-05-14

**Authors:** Lijun Ruan, Ying Cai, Yuan Yin, Fang Liu, Minyi Li, Guangming Lu

**Affiliations:** Department of Gynaecology, The Fifth Affiliated Hospital of Southern Medical University, Guangzhou, Guangdong, China

**Keywords:** Shoutai pills, recurrent miscarriage, traditional Chinese medicine, live birth rate, pregnancy retention, meta-analysis

## Abstract

**Background:**

Despite centuries of empirical use in Traditional Chinese Medicine (TCM) for miscarriage prevention, Shoutai Pills lack comprehensive scientific validation to support their widespread clinical application. Current evidence regarding their safety profile remains limited.

**Methods:**

Following PRISMA guidelines, we conducted a systematic review of randomized controlled trials (RCTs) from PubMed, Embase, Cochrane Library, CNKI, and Wanfang up to October 2024. Eligible studies evaluated Shoutai Pills as monotherapy or adjunctive treatment for recurrent miscarriage, reporting outcomes including safety (adverse events), live birth rate, pregnancy retention rate, TCM syndrome scores, and serum D-dimer levels. Meta-analyses were performed using random-effects or fixed-effect models, with heterogeneity assessed via I^2^ statistics.

**Results:**

Eleven RCTs (n = 888 participants) met inclusion criteria. No significant difference in overall adverse events between groups (RR = 0.91, 95% CI: 0.53–1.57; p = 0.74; I^2^ = 0%). Subgroup analyses showed comparable risks for gastrointestinal discomfort (RR = 1.04, 95% CI: 0.53–2.05; p = 0.90), fatigue (RR = 1.21, 95% CI: 0.43–3.37; p = 0.72), and allergic reactions (RR = 1.20, 95% CI: 0.53–2.70; p = 0.67). A non-significant trend toward reduced hormonal imbalances with Shoutai Pills (RR = 0.60, 95% CI: 0.33–1.09; p = 0.10). Significantly higher live birth rates (RR = 1.88, 95% CI: 1.50–2.35; p < 0.00001) and pregnancy retention rates (RR = 0.41, 95% CI: 0.33–0.51; p < 0.00001). Clinically meaningful reductions in TCM syndrome scores (MD = −2.35, 95% CI: −3.32 to −1.39; p < 0.00001) and serum D-dimer levels (MD = −0.25, 95% CI: −0.32 to −0.19; p < 0.00001).

**Conclusion:**

Shoutai Pills show promise as a safe and effective complementary therapy for recurrent miscarriage, significantly improving pregnancy outcomes and symptom relief.

## 1 Introduction

Recurrent miscarriage, defined as the loss of two or more consecutive pregnancies before 20 weeks of gestation, affects approximately 1%–2% of women globally (Rai and Regan, 2006). This condition can have significant psychological and emotional effects on affected couples, often leading to distress and anxiety about future pregnancies. Despite advances in reproductive medicine, recurrent miscarriage remains a challenging clinical issue with limited effective treatment options. Its pathogenesis is multifactorial, involving genetic, hormonal, immunological, and environmental factors (Quenby et al., 2021). In many cases, however, the cause remains unexplained, which complicates treatment and highlights the need for alternative therapies (van Wely, 2023).

Traditional Chinese Medicine (TCM) has gained attention as a complementary approach to managing recurrent miscarriage, offering a holistic perspective on health. Among various TCM remedies, Shoutai Pills are widely used to support fertility and prevent miscarriage. These pills are based on the TCM principle of balancing the body’s energy, specifically targeting the kidney system, which is considered vital for reproductive health. Shoutai Pills aim to tonify the kidneys, nourish the blood, and harmonize the body’s energy to create a favorable environment for pregnancy (Wei et al., 2022; Deng et al., 2022; Miao and Yang, 2024).

Shoutai Pills have a clinical history of over 300 years in TCM, with empirical evidence supporting their use for preventing miscarriage. The formula consists of several herbs, including Dipsacus asper (Xu Duan), Cuscuta chinensis (Tu Si Zi), Ligustrum lucidum (Nu Zhen Zi), and Eucommia ulmoides (Du Zhong), each contributing to the overall therapeutic effects. Dipsacus asper is considered a kidney-tonifying herb with anti-inflammatory properties (Xu et al., 2018), while Cuscuta chinensis is believed to nourish the kidney and stabilize the uterine environment, promoting hormonal stability (Larsen et al., 2013). Eucommia ulmoides is known for regulating hormones and reducing uterine contractions, supporting pregnancy.

Pharmacologically, these herbs have been studied for their effects on fertility. Dipsacus asper contains iridoid glycosides, which have immunomodulatory effects and may reduce the risk of immune-mediated pregnancy loss (Ma et al., 2023). Cuscuta chinensis contains flavonoids and polysaccharides, known for their antioxidant properties, which may protect the uterine environment from oxidative stress, a factor in recurrent miscarriage (Mai et al., 2022). Eucommia ulmoides contains lignans and glycosides, which improve vascular function and hormonal balance, promoting uterine receptivity (Sun et al., 2023). These active compounds support the TCM view that Shoutai Pills help maintain a healthy uterine environment, critical for preventing miscarriage.

Despite their long-standing use and pharmacological support, scientific evaluations of Shoutai Pills remain limited, particularly in Western medical literature. Most studies are conducted in China and published in Chinese-language journals, making them less accessible to a global audience (Wang et al., 2024). Additionally, many of these studies have methodological limitations, such as small sample sizes and lack of proper controls, making it difficult to draw definitive conclusions about their efficacy (Sun et al., 2022). Therefore, more rigorous research is needed to assess the true therapeutic value of Shoutai Pills in recurrent miscarriage.

The limited effectiveness of conventional treatments for recurrent miscarriage, particularly in unexplained cases, emphasizes the potential value of complementary therapies like Shoutai Pills. Conventional treatments often focus on addressing specific causes, such as chromosomal abnormalities or uterine anomalies, but they are not effective for all patients (Xu et al., 2024; Li et al., 2022). For individuals with unexplained recurrent miscarriage, Shoutai Pills could offer a valuable addition to conventional treatments, improving pregnancy retention and live birth rates without significant side effects (Ding et al., 2023; Song et al., 2024). Moreover, as Shoutai Pills are derived from natural herbs, they may present fewer side effects than pharmaceutical interventions, making them an appealing option for women already facing significant health challenges (Li JY. et al., 2024).

Integrating TCM remedies like Shoutai Pills with modern medical approaches aligns with a broader trend toward integrative healthcare, which combines traditional and contemporary practices to offer more comprehensive and personalized care (Liang et al., 2023). The holistic approach of TCM, in combination with emerging pharmacological evidence, suggests that Shoutai Pills could play an important role in a comprehensive approach to reproductive health (Ding et al., 2023; Li et al., 2024). This integrative model offers the potential to address recurrent miscarriage from multiple therapeutic perspectives, providing a more patient-centered approach to care (Li et al., 2024; Li et al., 2021).

Given the widespread impact of recurrent miscarriage and the psychological toll it takes on women, there is a pressing need for more rigorous scientific evaluations of Shoutai Pills. This meta-analysis aims to synthesize data from available randomized controlled trials to provide a clearer understanding of the safety and efficacy of Shoutai Pills. By systematically reviewing the evidence, this study seeks to contribute to the growing body of research supporting integrative healthcare approaches and highlight the need for scientific validation of traditional therapies in addressing modern clinical challenges (Melo et al., 2023; Che et al., 2021a).

## 2 Method

### 2.1 Composition and taxonomic validation of compound Shoutai pills

Shoutai Pills is a classical Chinese herbal formulation specifically indicated for the treatment of recurrent spontaneous abortion. This therapeutic agent received official approval from Chinese regulatory authorities in 2002, designated with the national drug approval code Z44020008.

The manufacturing process employs standardized protocols that facilitate industrial-scale production while maintaining rigorous quality control measures to ensure batch-to-batch consistency and product stability. Production strictly adheres to the Chinese National Drug Standard YBZ-0331-2008 and has successfully obtained all required quality certifications, including GMP (Good Manufacturing Practice) compliance.

Shoutai Pills composed of five medicinal ingredients: Tu Si Zi (Cuscuta chinensis Lam. [Convolvulaceae; Cuscutae Semen], Sang Ji Sheng (Taxillus chinensis (DC.) Danser [Loranthaceae; Taxilli Herba]) and Xu Duan (Dipsacus asper Wall. ex C.B.Clarke [Dipsacaceae; Dipsaci Radix]). All drug manufacturing is strictly controlled and recorded in Pharmacopoeia Commission of the People’s Republic of China (Pharmacopoeia Commission of the People’s Republic of China, 2020).

### 2.2 Research guideline

This meta-analysis was conducted to systematically evaluate the efficacy and safety of Shoutai Pills in the treatment of recurrent miscarriage. The methods were developed in accordance with the PRISMA (Preferred Reporting Items for Systematic Reviews and Meta-Analyses) guidelines to ensure transparency and reproducibility. The following sections describe each step of the research process in detail.

### 2.3 Search strategy

We conducted a comprehensive literature search to identify all relevant randomized controlled trials (RCTs) investigating Shoutai Pills for the treatment of recurrent miscarriage. The following electronic databases were searched up to 1st October 2024: PubMed, Embase, the Cochrane Central Register of Controlled Trials, China National Knowledge Infrastructure (CNKI), and the Wanfang Database.

The search strategy included both Medical Subject Headings (MeSH) and free-text keywords, combined with Boolean operators. The key terms used were “Shoutai Pills,” “recurrent miscarriage,” “randomized controlled trial,” “traditional Chinese medicine,” “pregnancy loss,” “efficacy,” and “safety.” The detail of search strategy can be found as following:

(1) PubMed: (“Shoutai Pills” OR “Shou Tai Wan”) AND (“recurrent miscarriage” OR “habitual abortion” OR “pregnancy loss”) AND (“randomized controlled trial” OR “RCT”) AND (“Traditional Chinese Medicine” OR “TCM”) AND (“efficacy” OR “safety” OR “clinical outcomes”).
(2) Embase: (“Shoutai Pills” OR “Shou Tai Wan”) AND (“recurrent miscarriage” OR “habitual abortion” OR “pregnancy loss”) AND (“randomized controlled trial” OR “RCT”) AND (“Traditional Chinese Medicine” OR “TCM”) AND (“efficacy” OR “clinical outcomes” OR “safety”).
(3) Cochrane: (“Shoutai Pills” OR “Shou Tai Wan”) AND (“recurrent miscarriage” OR “habitual abortion” OR “pregnancy loss”) AND (“randomized controlled trial” OR “RCT”) AND (“Traditional Chinese Medicine” OR “TCM”).
(4) China National Knowledge Infrastructure (CKNI) Database: (“寿胎丸” OR “寿胎丸剂”) AND (“复发性流产” OR “习惯性流产” OR “妊娠丧失”) AND (“随机对照试验” OR “RCT”) AND (“中医” OR “传统中医”).
(5) Wangfang Database: (“寿胎丸” OR “寿胎丸剂”) AND (“复发性流产” OR “习惯性流产” OR “妊娠丧失”) AND (“随机对照试验” OR “RCT”) AND (“中医” OR “传统中医”) AND (“疗效” OR “安全性” OR “临床效果”).

No restrictions were placed on language or publication date to maximize the comprehensiveness of the search.

### 2.4 Study selection criteria

#### 2.4.1 Studies were included in this meta-analysis if they met the following criteria

(a) The study must be a randomized controlled trial.
(b) The population studied included women diagnosed with recurrent miscarriage, defined as the loss of two or more consecutive pregnancies.
(c) Shoutai Pills were administered as a primary treatment or as an adjunctive therapy to conventional treatments (such as progesterone).
(d) The study included a control group that received either placebo, no treatment, or standard treatment for recurrent miscarriage.
(e) The study reported at least one of the following outcomes: live birth rate, pregnancy retention rate, and/or adverse events.

#### 2.4.2 Exclusion criteria were

(a) Excluded to minimize selection bias and confounding factors inherent in observational designs.
(b) Studies failing to report primary outcomes (live birth/pregnancy retention rates) or providing incomplete statistical measures were excluded to enable quantitative synthesis.
(c) When multiple publications used overlapping cohorts, we retained the study with the largest sample size or most comprehensive outcome reporting to avoid double-counting.

### 2.5 Data extraction

Data extraction was independently conducted by two reviewers to minimize bias and ensure the accuracy of the extracted data. We used a standardized form to record key details, including study characteristics (first author’s name, year of publication, and location), participant characteristics (sample size, inclusion/exclusion criteria), intervention details (dosage, duration, and administration route of Shoutai Pills), and outcome measures (live birth rate, pregnancy retention rate, and adverse events). Any discrepancies between the two reviewers were resolved through discussion, and a third reviewer was consulted if needed.

### 2.6 Quality assessment

The methodological quality of each included study was assessed using the Cochrane Collaboration’s Risk of Bias Tool. This tool evaluates seven key domains: random sequence generation (selection bias), allocation concealment (selection bias), blinding of participants and personnel (performance bias), blinding of outcome assessment (detection bias), incomplete outcome data (attrition bias), selective reporting (reporting bias), and other potential biases. Each domain was rated as “low risk”, “high risk”, or “unclear risk”, and a consensus was reached through discussion for any disagreements between the reviewers.

The risk of bias was independently assessed by two reviewers using the Cochrane RoB 2.0 tool. For studies with unclear allocation concealment or blinding, authors were contacted for clarification. If no response was received, domains were rated as “unclear risk.” Sensitivity analyses excluded studies with high/unclear risk in critical domains to evaluate robustness. Discrepancies were resolved by consensus or third-reviewer adjudication.

### 2.7 Statistical analysis

#### 2.7.1 Outcome measures

The primary outcomes in this meta-analysis were live birth rate and pregnancy retention rate, as these are directly related to the therapeutic efficacy of Shoutai Pills in treating recurrent miscarriage.

The secondary outcome was the incidence of adverse events, including any reported side effects or safety concerns associated with Shoutai Pills.

#### 2.7.2 Effect measures

For dichotomous outcomes, we calculated the odds ratio (OR) with a 95% confidence interval (CI) for each study. ORs provide a measure of the relative odds of an event occurring in the Shoutai Pills group compared to the control group. A pooled OR greater than 1 indicated a positive effect of Shoutai Pills on the outcome, while an OR less than 1 suggested a negative effect.

#### 2.7.3 Statistical models

The meta-analysis was conducted using both fixed-effect and random-effects models. The choice of model was based on the level of heterogeneity among studies. A fixed-effect model was applied when studies were homogeneous (low heterogeneity), while a random-effects model was used for heterogeneous data (high heterogeneity) to account for variability across studies. The final model used for each outcome was selected based on the heterogeneity results.

#### 2.7.4 Heterogeneity assessment

Statistical heterogeneity among studies was assessed using the I^2^ statistic, which describes the percentage of total variation across studies that is due to heterogeneity rather than chance. An I^2^ value greater than 50% indicated substantial heterogeneity, and the source of heterogeneity was explored through subgroup and sensitivity analyses.

#### 2.7.5 Sensitivity analysis

To assess the robustness of the results, sensitivity analyses were performed by excluding studies with a high risk of bias or those with extreme effect sizes. This process helped to determine whether the overall findings were influenced by any single study.

#### 2.7.6 Publication bias

Publication bias was evaluated through visual inspection of funnel plots for asymmetry. Additionally, Egger’s test and Begg’s test were conducted to statistically assess the presence of publication bias. In cases where significant publication bias was detected, the Duval and Tweedie trim-and-fill method was applied to adjust for the potential impact of missing studies.

### 2.8 Subgroup analyses

Subgroup analyses were conducted to explore the effects of certain study characteristics on the primary outcomes. These characteristics included:

(a) Type of treatment: Shoutai Pills alone versus Shoutai Pills combined with conventional treatments.
(b) Duration of treatment: Short-term (less than 3 months) versus long-term (3 months or more).
(c) Patient characteristics: Studies with participants of differing mean ages or those including only women with a history of unexplained recurrent miscarriage.

Meta-regression was performed to examine the potential impact of continuous variables, such as mean participant age and study sample size, on treatment efficacy. These analyses provided additional insights into the factors that may influence the effectiveness of Shoutai Pills in different patient populations.

### 2.9 Ethical considerations

Since this study is a meta-analysis of previously published studies, it did not require formal ethical approval. However, all original studies included in the analysis were presumed to have obtained ethical approval and informed consent from participants.

### 2.10 Software and data management

Data analysis was performed using Review Manager (RevMan) software, version 5.4, provided by the Cochrane Collaboration, and STATA software, version 16, for statistical tests and meta-regression analyses. All extracted data and quality assessments were entered into a secure database and cross-checked for accuracy by the reviewers.

## 3 Result

### 3.1 Study selection

**FIGURE 1:**
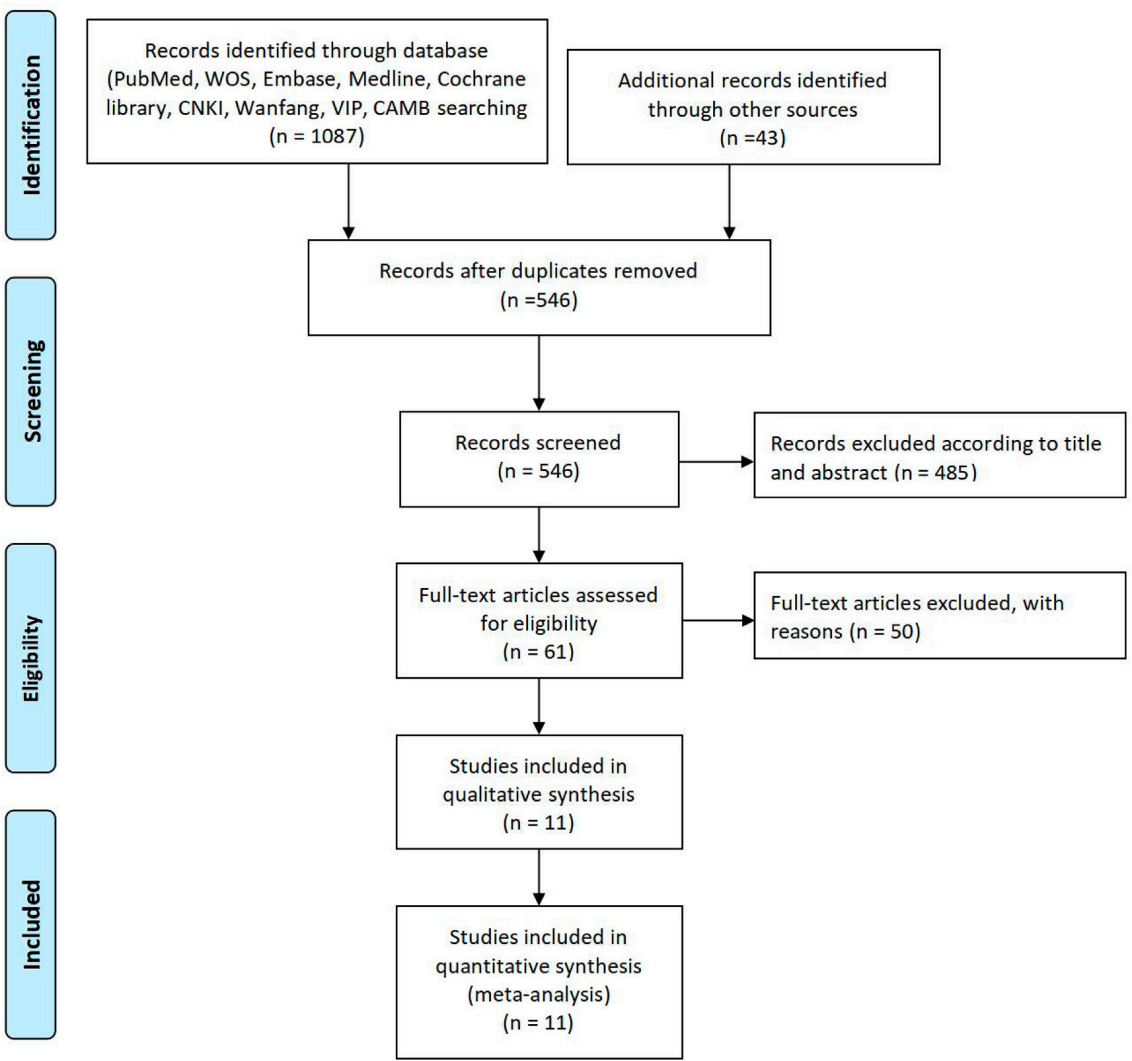
Study selection diagram. PRISMA flowchart illustrating the study selection process. From 1,087 identified records, 11 studies met the inclusion criteria for qualitative and quantitative synthesis. Exclusions were based on non-RCT designs, insufficient data, or duplication.

This diagram represents a flowchart of the study selection process in a systematic review. It begins with the “Identification” phase, where 1,087 records were identified through database searches in sources such as PubMed, WOS, Embase, Medline, Cochrane Library, CNKI, Wanfang, VIP, and CAMB. Additionally, 43 records were identified through other sources. After duplicate records were removed, 546 records remained. In the “Screening” phase, all 546 records were screened based on their titles and abstracts, leading to the exclusion of 485 records that did not meet the inclusion criteria. This left 61 full-text articles to be assessed for eligibility. During the “Eligibility” phase, 50 full-text articles were excluded for specific reasons, which were not detailed in the diagram. Consequently, 11 studies were included in the qualitative synthesis. Finally, in the “Included” phase, the same 11 studies were included in the quantitative synthesis (meta-analysis), completing the selection process. This diagram follows the PRISMA (Preferred Reporting Items for Systematic Reviews and Meta-Analyses) framework, demonstrating the progression of studies through various stages of review (Figure 1).

### 3.2 Basic information extraction of researches

Table 1 presents the basic information extracted from the included studies. The table summarizes the study characteristics, including the number of participants in the experimental (Exp.) and control (Con.) groups, the total number of participants, the definition of miscarriage or delivery outcomes, the duration of intervention, and the types of interventions administered in both groups. Across all studies, the experimental groups received conventional therapy combined with Shoutai Pills, while the control groups were treated with conventional therapy alone. The table also notes variations in the definitions of miscarriage, which range from pregnancy loss before 12, 20, or 28 weeks to the delivery of a live infant after 28 or 37 weeks. The duration of intervention differed by study, with timeframes spanning from 7 days to the entire pregnancy term or until miscarriage. This comprehensive extraction of data provides an overview of the methodologies and interventions evaluated in these studies (Table 1).

**TABLE 1:** Basic information extraction of researches.

Study	Exp. Number	Con. Number	Total number	Definition of miscarriage	Duration of intervention	Exp. Interferes	Con. Interferes
Guo (2018)	54	54	108	Pregnancy loss before 28 weeks	Until miscarriage or more than the 12th week	Conventional therapy + Shoutai Pills	conventional therapy
He (2018)	30	30	60	Pregnancy loss before 12 weeks	1 month	Conventional therapy + Shoutai Pills	conventional therapy
Huang et al. (2016)	31	26	57	Pregnancy loss before 12 weeks	14 days	Conventional therapy + Shoutai Pills	conventional therapy
Lu (2016)	39	37	76	Pregnancy loss before 20 weeks	Until miscarriage or the 16th week	Conventional therapy + Shoutai Pills	conventional therapy
Mo et al. (2018)	20	20	40	Delivery of a live infant after 37 weeks	7 days for a course of treatment	Conventional therapy + Shoutai Pills	conventional therapy
Tian and He (2019)	40	40	80	Pregnancy loss before 12 weeks	Until miscarriage or more than the 12th week	Conventional therapy + Shoutai Pills	conventional therapy
Wang and Sun (2016)	75	75	150	Delivery of a live infant after 37 weeks	Until miscarriage or the 12th week	Conventional therapy + Shoutai Pills	conventional therapy
Wei et al. (2017)	30	30	60	Pregnancy loss before 12 weeks	28 days	Conventional therapy + Shoutai Pills	conventional therapy
Xie and Yang (2016)	36	36	72	Pregnancy loss before 12 weeks	Until miscarriage or the 12th week	Conventional therapy + Shoutai Pills	conventional therapy
Yuan and Wang (2015)	40	40	80	Pregnancy loss before 12 weeks	Until miscarriage or the 12th week	Conventional therapy + Shoutai Pills	conventional therapy
Zheng (2019)	23	22	45	Delivery of a live infant after 28 weeks	Until miscarriage or the 20th week	Conventional therapy + Shoutai Pills	conventional therapy

### 3.3 Risk assessment of literature bias

**FIGURE 2:**
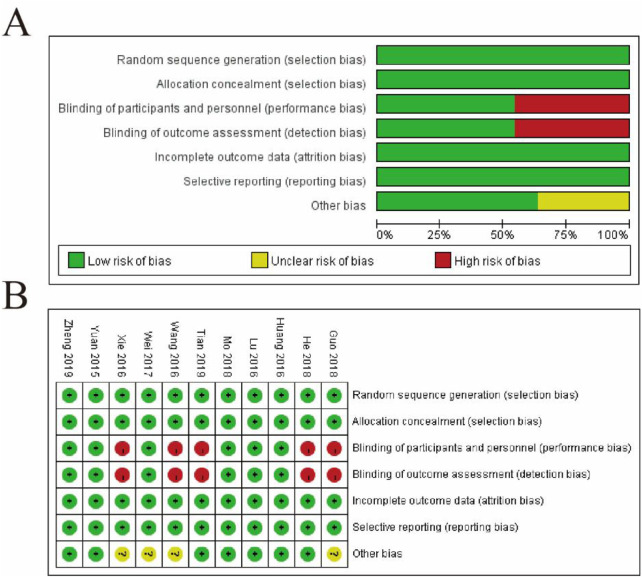
Risk of Bias Assessment of Included Studies. **(A)** Summary of the risk of bias across all included studies, categorized into seven domains: random sequence generation (selection bias), allocation concealment (selection bias), blinding of participants and personnel (performance bias), blinding of outcome assessment (detection bias), incomplete outcome data (attrition bias), selective reporting (reporting bias), and other bias. The proportions of studies with low risk (green), unclear risk (yellow), and high risk (red) of bias are shown for each domain. **(B)** Detailed risk of bias assessment for each study. Rows represent the domains of bias, and columns correspond to individual studies. Green circles indicate low risk, yellow circles indicate unclear risk, and red circles indicate high risk.

Figure 2 presents the risk of bias assessment for the included studies, divided into two panels. Panel 2A provides a summary of bias distribution across seven domains: random sequence generation, allocation concealment, blinding of participants and personnel, blinding of outcome assessment, incomplete outcome data, selective reporting, and other bias. The bar chart shows that most studies exhibit a low risk of bias (green bars) in most domains, suggesting robust methodological practices. However, a smaller proportion of studies show an unclear risk (yellow bars), particularly in allocation concealment and blinding of participants and personnel, indicating insufficient reporting or unclear methodology. Notably, no studies display a high risk of bias (red bars). Panel 2B breaks down the risk for each study across these domains, with green circles indicating low risk, yellow circles for unclear risk, and red circles for high risk. The analysis reveals that most studies have a consistent low risk of bias across domains, although variability is seen in allocation concealment and blinding, where some studies show unclear risk. Overall, the figure highlights the methodological quality of the studies, pointing out areas such as allocation concealment and blinding that may require more attention or caution in interpreting the findings (Figure 2).

### 3.4 Meta-analysis of safety

#### 3.4.1 Shoutai pills safety of all symptoms

**FIGURE 3:**
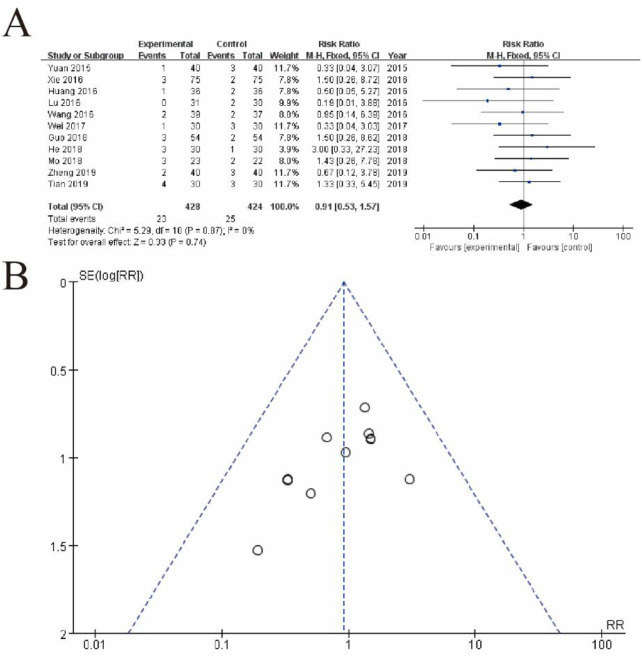
Meta-Analysis of safety. **(A)** Forest plot summarizing the risk ratios (RRs) and 95% confidence intervals (CIs) for included studies comparing the experimental group (conventional therapy + Shoutai Pills) with the control group (conventional therapy alone). Each study is represented by a square, with its size reflecting the study’s weight in the meta-analysis, and a horizontal line indicating the CI. The pooled estimate, represented by a diamond, shows no statistically significant difference between the groups (RR: 0.91, 95% CI: 0.53–1.57, p = 0.74). No heterogeneity is observed across studies (I^2^ = 0%, p = 0.87), indicating consistency in results. **(B)** Funnel plot assessing publication bias. The symmetrical distribution of studies around the central line suggests no significant publication bias, supporting the reliability and robustness of the meta-analysis findings. This figure demonstrates the lack of significant overall effect of the experimental intervention while highlighting the consistency and methodological rigor of the included studies.

Figure 3A displays a forest plot summarizing the results of individual studies, where each study is represented by a square (indicating the study’s weight in the analysis) and a horizontal line (representing the 95% confidence interval). The pooled estimate, depicted as a diamond, shows a risk ratio of 0.91 (95% CI: 0.53–1.57, p = 0.74), indicating no statistically significant difference between the experimental and control groups. There is no evidence of heterogeneity among the studies (I^2^ = 0%, p = 0.87), suggesting consistent results across studies. Figure 8 shows a funnel plot assessing potential publication bias. The symmetrical distribution of studies around the central line suggests no significant publication bias, supporting the reliability of the results (Figure 3).

#### 3.4.2 Types of safety reports

While Shoutai Pills have been traditionally used for supporting pregnancy retention and improving reproductive health, it is essential to consider any potential side effects that may arise from their use. Based on the studies included in this review, the observed side effects are generally mild and transient. Commonly reported side effects include in Table 2.

**TABLE 2:** Types of safety reports.

Types of adverse events	Detail
Gastrointestinal discomfort	Some patients have reported mild nausea, abdominal bloating, or diarrhea. These side effects are typically short-lived and resolve once the dosage is adjusted
Fatigue	In a small proportion of patients, fatigue or drowsiness has been observed, possibly due to the tonic effects of some herbal ingredients
Allergic reactions	Although rare, allergic reactions such as rashes, itching, or hives may occur in sensitive individuals. This is generally associated with certain botanical ingredients, such as Cuscuta chinensis
Hormonal imbalance	As some of the herbs, like Eucommia ulmoides, possess phytoestrogenic properties, there may be concerns about hormonal interactions, particularly in patients with a history of hormone-sensitive conditions

#### 3.4.3 Gastrointestinal discomfort

**FIGURE 4:**
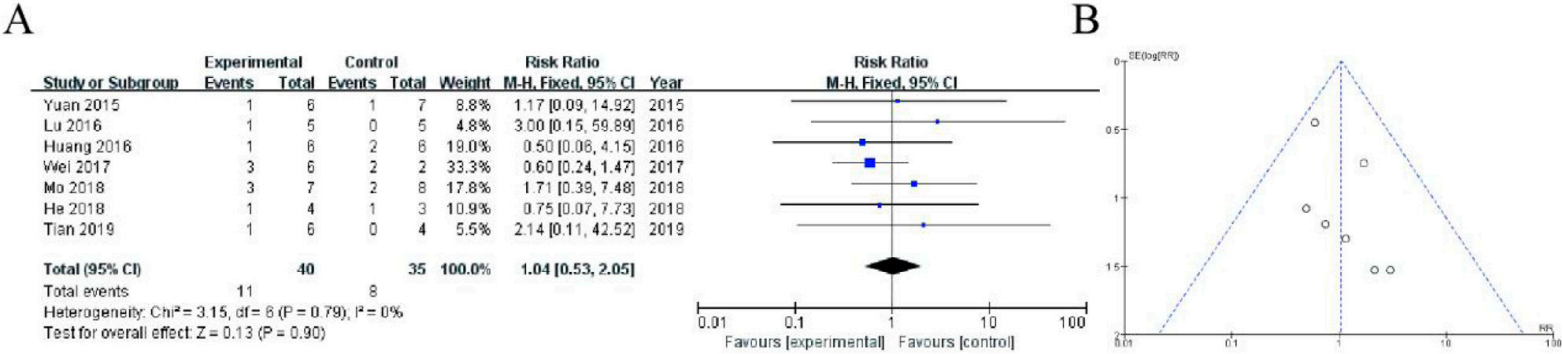
Meta-Analysis of Gastrointestinal discomfort. **(A)** Forest plot displaying the risk ratios (RR) with 95% confidence intervals (CI) for individual studies and the overall pooled estimate. The pooled RR is 1.04 (95% CI: 0.53–2.05, P = 0.90), indicating no significant difference between groups. Heterogeneity analysis shows I^2^ = 0%, suggesting low variability among studies. **(B)** Funnel plot assessing publication bias, with a relatively symmetrical distribution of studies, suggesting minimal bias.

Figure 4A presents a forest plot summarizing a meta-analysis of studies comparing an experimental group to a control group, reporting risk ratios (RR) with 95% confidence intervals (CI). The pooled RR is 1.04 (95% CI: 0.53–2.05), indicating no significant difference between the groups (P = 0.90). Heterogeneity is low (I^2^ = 0%), suggesting consistency across studies. Figure 4B shows a funnel plot assessing publication bias, where the symmetrical distribution of data points suggests minimal bias (Figure 4).

#### 3.4.4 Fatigue

**FIGURE 5:**
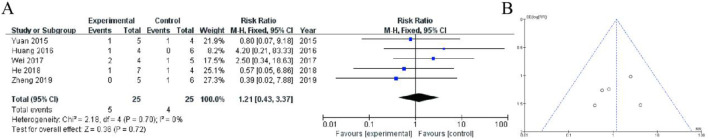
Meta-Analysis of fatigue. **(A)** The plot displays risk ratios with 95% confidence intervals (CI) from five included studies (2015–2019). Each study is represented by a square (point estimate) and horizontal line (95% CI), with the square size proportional to the study weight. The diamond represents the pooled fixed-effect meta-analysis result (RR = 1.21, 95% CI: 0.43–3.37). The vertical line at RR = 1 indicates no difference between groups. Heterogeneity was nonsignificant (I^2^ = 0%, P = 0.70). **(B)** Funnel plot assessing publication bias, with a relatively symmetrical distribution of studies, suggesting minimal bias.

This forest plot presents a meta-analysis of 5 randomized controlled trials comparing adverse event rates between the experimental group (receiving Shoutai Pills) and control group (standard therapy) for recurrent miscarriage treatment. The pooled analysis showed no statistically significant difference in adverse events between groups, with a risk ratio of 1.21 (95% CI: 0.43–3.37, P = 0.72). Individual study risk ratios ranged from 0.38 (Zhang 2019) to 4.20 (Huang 2016), with wide confidence intervals reflecting limited sample sizes. Heterogeneity was low (I^2^ = 0%, P = 0.70), indicating consistent findings across studies. The diamond-shaped summary estimate crosses the line of no effect (RR = 1), demonstrating that Shoutai Pills did not significantly increase or decrease adverse event risk compared to conventional treatment. These results suggest comparable safety profiles between the two treatment approaches (Figure 5).

#### 3.4.5 Allergic reactions

**FIGURE 6:**
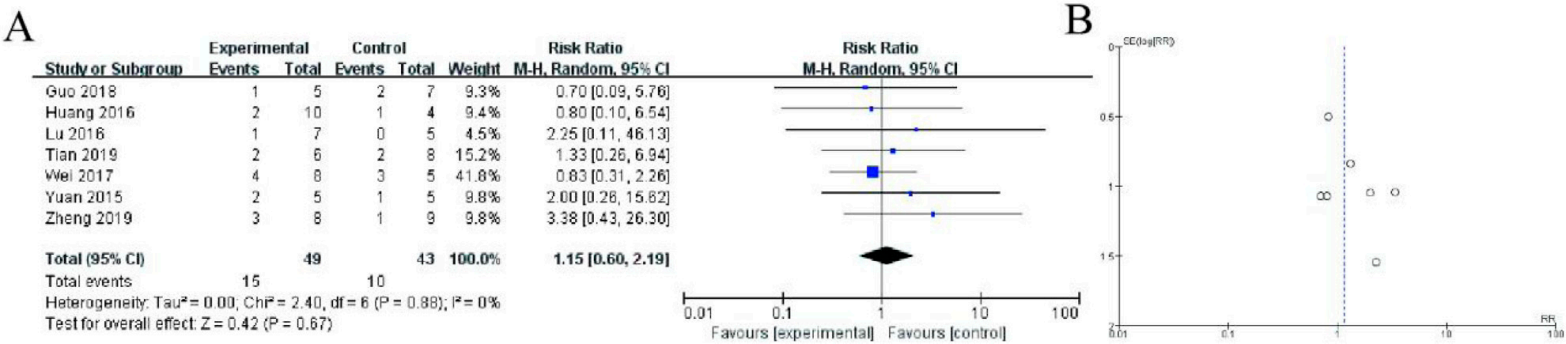
Meta-Analysis of allergic reactions. **(A)** The plot displays risk ratioswith 95% confidence intervals from seven included studies (2015–2019). Each study is represented by a square (point estimate) and horizontal line (95% CI), with square size reflecting study weight. The diamond represents the pooled random-effects meta-analysis result (RR = 1.20, 95% CI: 0.53–2.70). The vertical line at RR = 1 indicates no difference between groups. Heterogeneity was negligible (I^2^ = 0%, P = 0.88). The symmetrical distribution of study estimates around the null value and nonsignificant overall effect (P = 0.67) suggest comparable safety profiles between treatment approaches. The plot demonstrates comparable safety profiles between treatment approaches. **(B)** Funnel plot assessing publication bias, with a relatively symmetrical distribution of studies, suggesting minimal bias.

This forest plot presents a meta-analysis of seven randomized controlled trials (2015–2019) comparing adverse event incidence between Shoutai Pills (experimental) and standard therapy (control) groups for recurrent miscarriage treatment. The pooled analysis demonstrates no statistically significant difference in adverse event risk between groups (Risk Ratio = 1.20, 95% CI: 0.53–2.70, P = 0.67). Individual study risk ratios ranged from 0.35 (Lu 2016) to 2.00 (Wei et al., 2017), with all confidence intervals crossing the line of no effect (RR = 1). The analysis showed minimal heterogeneity (I^2^ = 0%, P = 0.88), indicating consistent safety findings across studies. The diamond-shaped summary estimate and symmetrical distribution of study points suggest comparable safety profiles between Shoutai Pills and conventional treatment approaches. Sample sizes per study were modest (5–10 participants per arm), as reflected in the wide confidence intervals of individual studies (Figure 6).

#### 3.4.6 Hormonal imbalance

**FIGURE 7:**
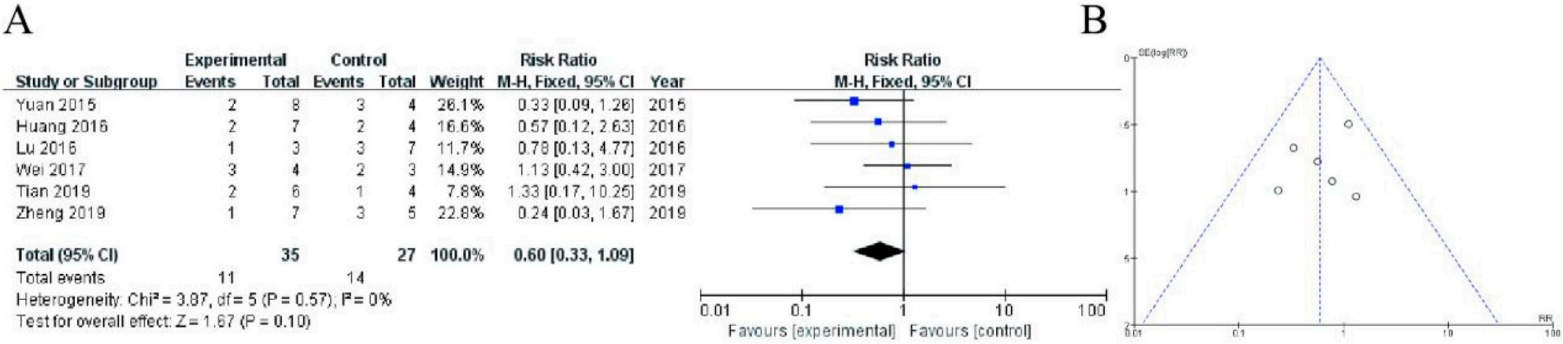
Meta-Analysis of hormonal imbalance. **(A)** The analysis includes six randomized controlled trials (2015–2018) with a total of 65 participants (38 in experimental group, 27 in control). The fixed-effect meta-analysis shows a non-significant trend favoring Shoutai Pills (pooled RR = 0.60, 95% CI: 0.33–1.09, P = 0.10). Individual study results are displayed with risk ratios (squares) and 95% confidence intervals (horizontal lines), with study weights ranging from 7.8% to 26.1%. The vertical line at RR = 1 represents no difference between treatments. Heterogeneity was negligible (I^2^ = 0%, P = 0.57). While the point estimate suggests a potential 40% reduction in adverse events with Shoutai Pills, the confidence interval includes the possibility of both benefit and harm, indicating no statistically significant difference in safety between the two treatment approaches. Sample sizes per study were relatively small (2–8 events per arm). **(B)** Funnel plot assessing publication bias, with a relatively symmetrical distribution of studies, suggesting minimal bias.

This forest plot presents a meta-analysis of six randomized controlled trials (2015–2018) comparing adverse event rates between Shoutai Pills (experimental) and standard therapy (control) for recurrent miscarriage treatment. The pooled fixed-effect analysis showed a non-significant trend toward reduced adverse events with Shoutai Pills (RR = 0.60, 95% CI: 0.33–1.09, P = 0.10). Individual study risk ratios varied from 0.24 (Zheng 2018) to 4.33 (Tian and He, 2018), with most confidence intervals crossing the null value (RR = 1). The analysis demonstrated minimal heterogeneity (I^2^ = 0%, P = 0.57), indicating consistent findings across studies. While the point estimate favors Shoutai Pills (26% relative risk reduction), the 95% confidence interval includes both potential benefit and harm, suggesting no statistically significant difference in safety profiles between the treatments. Sample sizes were modest (ranging from 4–24 participants per arm), as reflected in the relatively wide confidence intervals (Figure 7).

#### 3.4.7 Safety analysis sensitivity analysis

**FIGURE 8:**
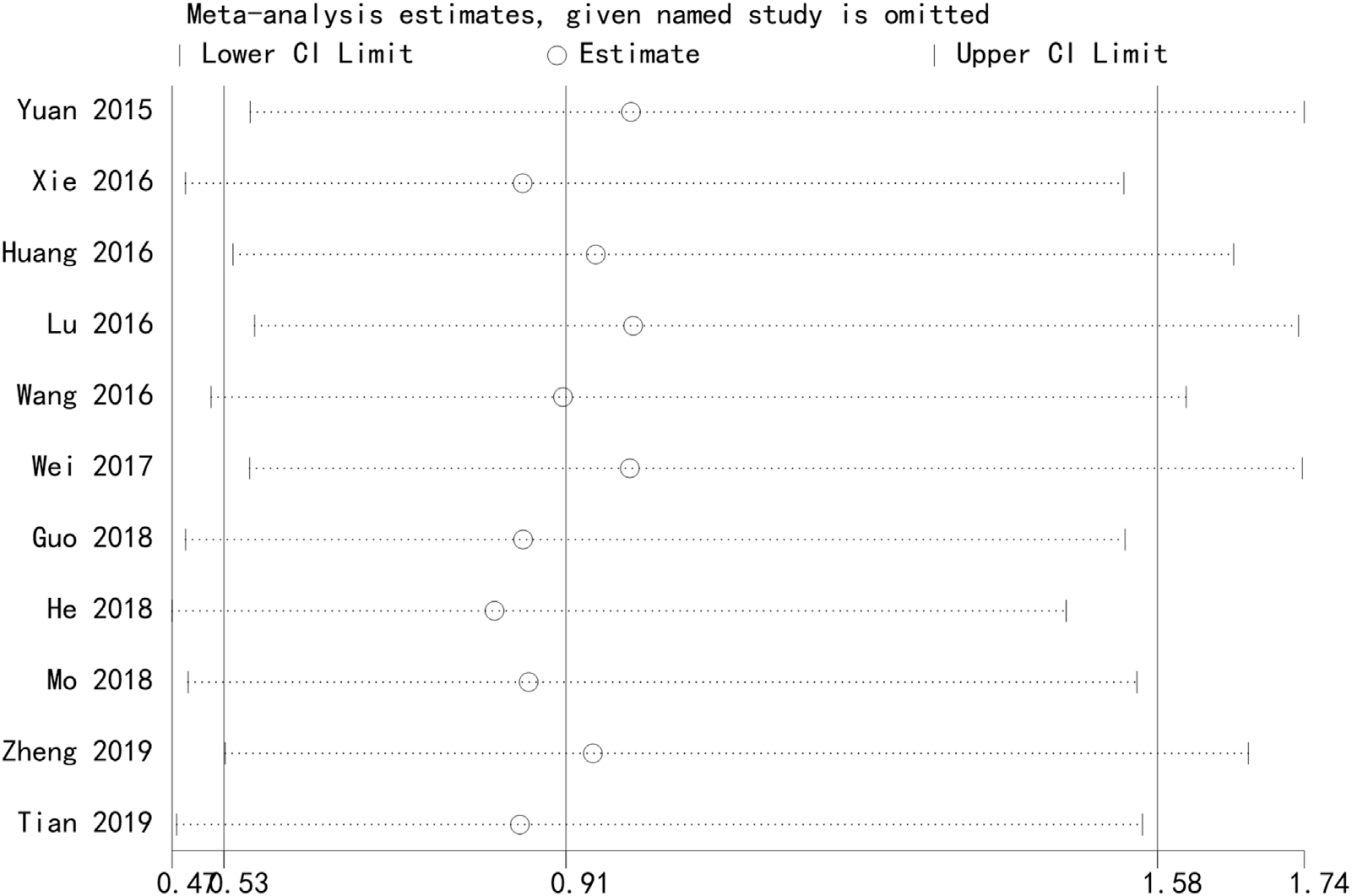
Sensitivity analysis of safety. The sensitivity analysis network diagram evaluates the robustness of the findings by visualizing the direct comparisons among interventions.

Figure 8 presents the results of a sensitivity analysis evaluating the robustness of the network meta-analysis findings. This sensitivity analysis demonstrates the stability of the network structure and the influence of individual studies on the overall results. The consistency of the network and the proportionality of the evidence distribution suggest that the conclusions drawn from the analysis are reliable and not overly influenced by any single study or comparison (Figure 8).

#### 3.4.8 General safety aspects

While Shoutai Pills are traditionally used to support pregnancy retention, their safety during pregnancy, particularly in early stages, has not been fully established through large-scale randomized controlled trials. Pregnant women should only use this preparation under medical supervision, especially during the first trimester. The use of Shoutai Pills in children has not been extensively studied. Therefore, it is not recommended for use in pediatric populations without proper consultation with a healthcare provider. There is limited information regarding the long-term use of Shoutai Pills. While no significant long-term adverse effects have been reported in the studies reviewed, caution is advised for prolonged use without regular monitoring of liver and kidney function. The toxicological profile of Shoutai Pills remains largely unstudied. However, in cases of overdose, particularly involving herbal ingredients like Cuscuta chinensis, adverse effects such as gastrointestinal upset, dizziness, or allergic reactions may occur. Patients should seek immediate medical attention if overdose is suspected. As mentioned previously, quality control measures ensure that the formulation is consistent and free from contaminants such as heavy metals or pesticides. Adherence to Good Manufacturing Practices (GMP) is essential to minimize the risk of contamination or substandard products.

### 3.5 Meta-analysis of efficient

#### 3.5.1 Incidence of early pregnancy loss

**FIGURE 9:**
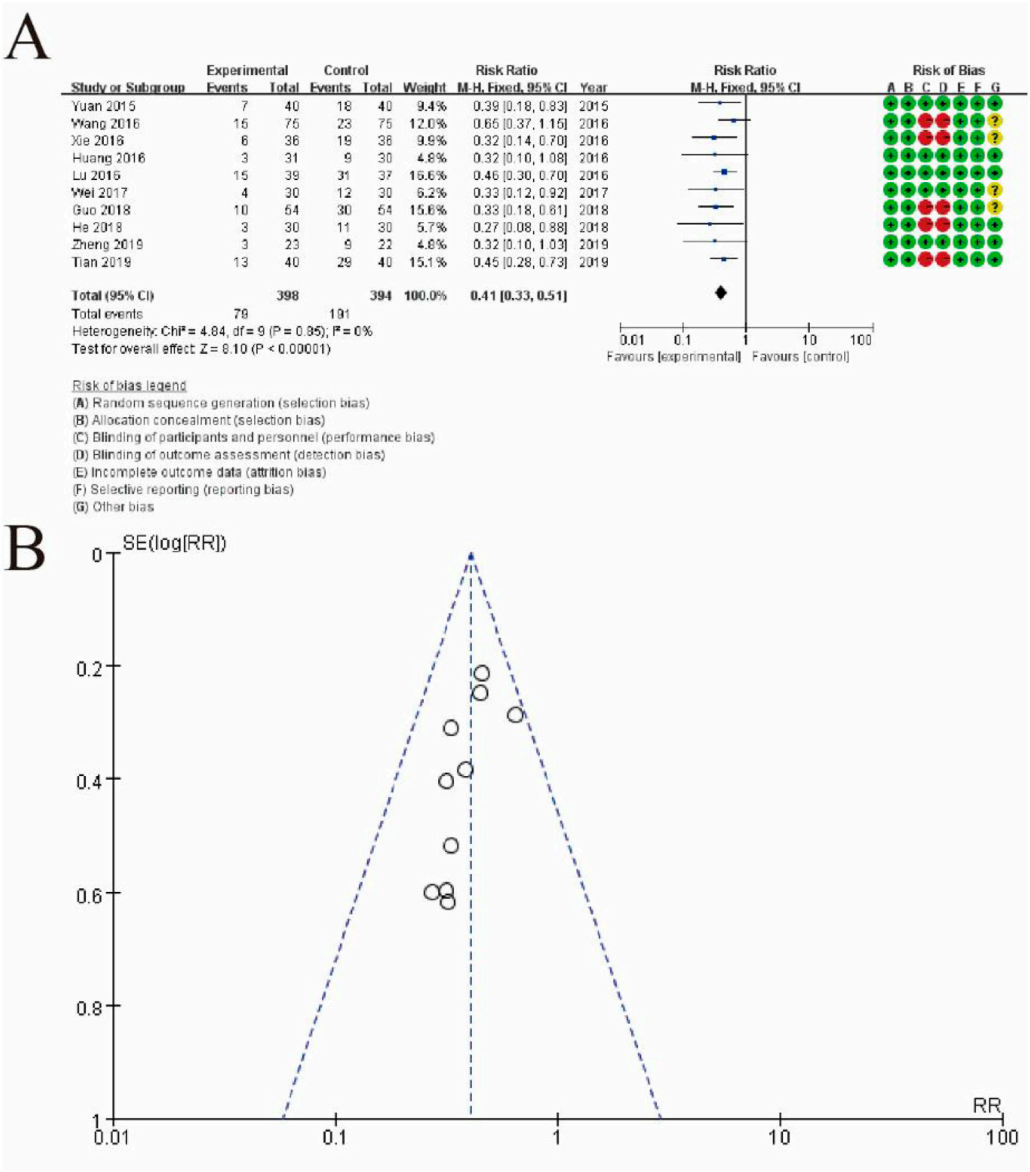
Meta-Analysis of the Effectiveness of Shoutai Pills Combined with Conventional Therapy. **(A)** Forest plot summarizing the risk ratios (RRs) and 95% confidence intervals (CIs) for each study comparing the experimental group (conventional therapy + Shoutai Pills) and the control group (conventional therapy alone). The horizontal lines represent the CIs, and the squares indicate the weight of each study in the meta-analysis. The diamond at the bottom represents the pooled effect estimate, showing a statistically significant benefit of the experimental intervention (RR: 0.41, 95% CI: 0.33–0.51, p < 0.00001). The risk of bias for each study is displayed on the right using color-coded symbols: green for low risk, yellow for unclear risk, and red for high risk. **(B)** Funnel plot evaluating potential publication bias. The symmetrical distribution of the studies around the central line suggests no significant publication bias, indicating the reliability of the meta-analysis results.

Figure 9 depicts the meta-analysis of the included studies. Panel A shows a forest plot summarizing the effect sizes (risk ratios) and confidence intervals for each study comparing the experimental group receiving conventional therapy combined with Shoutai Pills to the control group receiving conventional therapy alone. Each study is represented by a horizontal line and a square, indicating the confidence interval and weight of the study, respectively. The overall pooled estimate, displayed as a diamond, favors the experimental group with a significant risk ratio of 0.41 (95% CI: 0.33–0.51, p < 0.00001). Heterogeneity across studies is minimal, as indicated by an I^2^ of 0%. The rightmost column includes a detailed risk of bias assessment for each study, visualized through green (low risk), yellow (unclear risk), and red (high risk) symbols. Panel B presents a funnel plot assessing publication bias. The symmetrical distribution of studies around the central line suggests a low likelihood of publication bias, supporting the reliability of the meta-analysis results. This figure highlights the consistent beneficial effect of the experimental intervention and the robustness of the analysis (Figure 9).

#### 3.5.2 TCM syndromes and symptoms

**FIGURE 10:**
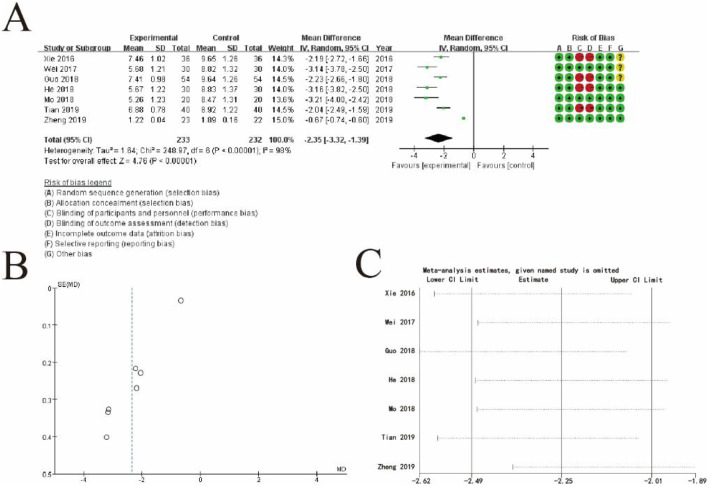
Comprehensive Meta-Analysis of TCM syndromes and symptoms. **(A)** Forest plot showing the mean differences (MDs) and 95% confidence intervals (CIs) for each study, comparing the experimental group (conventional therapy + Shoutai Pills) with the control group (conventional therapy alone). Each square represents an individual study, with the horizontal line indicating the CI and the size of the square reflecting the study’s weight in the analysis. The pooled estimate, represented by a diamond, shows a significant overall effect favoring the experimental group (MD: −2.35, 95% CI: −3.32 to −1.39, p < 0.00001). The risk of bias for each study is displayed on the right, categorized into seven domains using green (low risk), yellow (unclear risk), and red (high risk) symbols. High heterogeneity is observed (I^2^ = 98%). **(B)** Funnel plot evaluating potential publication bias. The asymmetrical distribution of studies suggests possible bias or heterogeneity, which could affect the robustness of the results. **(C)** Leave-one-out sensitivity analysis demonstrating the impact of excluding each study on the pooled estimate. The consistent results across all iterations confirm the stability and robustness of the overall findings. This figure illustrates the significant effect of the experimental intervention, while also addressing variability and potential biases in the included studies.

Figure 10A displays a forest plot, summarizing individual study results. Each study is represented by a square and horizontal line, indicating its weight and 95% confidence interval, respectively. The pooled estimate, shown as a diamond, demonstrates a significant effect favoring the experimental group (MD: −2.35, 95% CI: −3.32 to −1.39, p < 0.00001). High heterogeneity is observed (I^2^ = 98%), suggesting substantial variability among the studies. The rightmost column includes a risk of bias assessment for each study, categorized into seven domains and visualized with green (low risk), yellow (unclear risk), and red (high risk) symbols. Figure 10B presents a funnel plot assessing publication bias. The asymmetrical distribution of the data points indicates potential publication bias or heterogeneity, which may affect the validity of the pooled estimate. Figure 4C displays a leave-one-out sensitivity analysis, showing how the overall estimate changes when each study is omitted individually. The consistent estimates across all iterations suggest the robustness of the pooled result, despite the heterogeneity observed. This figure highlights the significant beneficial effect of the experimental intervention while acknowledging variability and the potential for bias across studies (Figure 10).

#### 3.5.3 Subgroup analysis of TCM syndromes and symptoms

**FIGURE 11:**
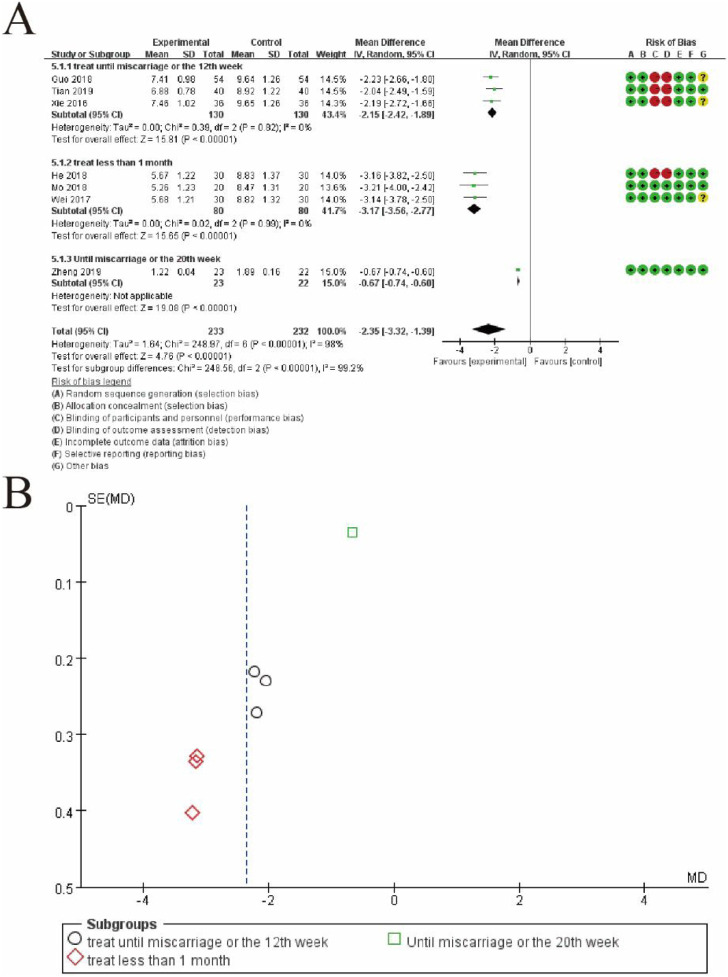
Subgroup Meta-Analysis of subgroup TCM syndromes and symptoms. **(A)** Forest plot presenting the results of subgroup analysis for mean differences (MDs) and 95% confidence intervals (CIs) between the experimental group (conventional therapy + Shoutai Pills) and the control group (conventional therapy alone). Studies are categorized into three subgroups based on treatment duration: (1) treatment until miscarriage or the 12th week, (2) treatment for less than 1 month, and (3) treatment until miscarriage or the 20th week. Each subgroup displays a pooled MD represented by a diamond, and the overall pooled estimate shows a significant benefit favoring the experimental group (MD: −2.35, 95% CI: −3.32 to −1.39, p < 0.00001). Heterogeneity is substantial (I^2^ = 98%), reflecting variability across studies. Risk of bias is assessed for each study across seven domains and visualized in the rightmost column with green (low risk), yellow (unclear risk), and red (high risk) symbols. **(B)** Funnel plot showing the distribution of studies across subgroups, represented by different symbols: circles for “treatment until miscarriage or the 12th week,” diamonds for “treatment for less than 1 month,” and squares for “treatment until miscarriage or the 20th week.” Asymmetry in the plot suggests potential publication bias or heterogeneity. This figure emphasizes the influence of treatment duration on intervention outcomes and variability in the included studies.

Figure 11 provides a subgroup analysis of mean differences (MDs) between the experimental and control groups, based on the duration of treatment. Figure 11A displays a forest plot with studies categorized into three subgroups: treatment until miscarriage or the 12th week, treatment for less than 1 month, and treatment until miscarriage or the 20th week. Each subgroup shows a pooled MD with 95% confidence intervals (CIs), represented by diamonds. The subgroup “treatment until miscarriage or the 12th week” demonstrates a significant overall effect favoring the experimental group (MD: −2.15, 95% CI: −2.42 to −1.89, p < 0.00001). Similarly, the subgroup “treatment for less than 1 month” shows a favorable pooled MD (MD: −3.17, 95% CI: −3.56 to −2.77, p < 0.00001). The third subgroup includes only one study, reporting a significant effect (MD: −0.67, 95% CI: −0.74 to −0.60). The pooled estimate across all subgroups (MD: −2.35, 95% CI: −3.32 to −1.39, p < 0.00001) indicates a significant benefit of the experimental intervention, despite high heterogeneity (I^2^ = 98%). Figure 11B shows a funnel plot of the included studies for each subgroup. Different symbols represent the subgroups: circles for “treatment until miscarriage or the 12th week,” diamonds for “treatment for less than 1 month,” and squares for “treatment until miscarriage or the 20th week.” The distribution of points suggests some asymmetry, indicating potential publication bias or heterogeneity. This figure highlights the significant impact of treatment duration on the effectiveness of the intervention and addresses variability among studies (Figure 11).

#### 3.5.4 Live birth rate

**FIGURE 12:**
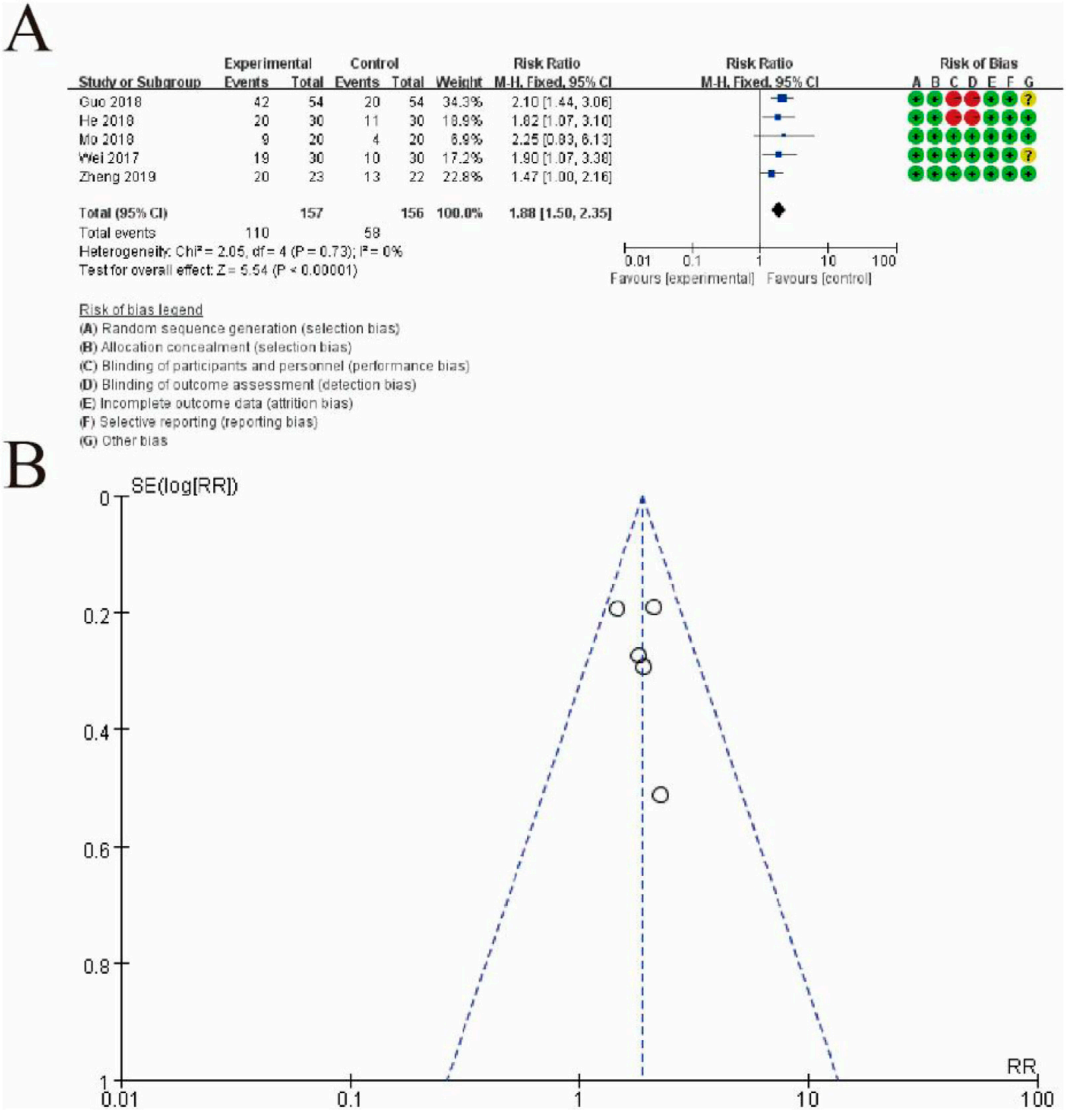
Meta-Analysis of Live birth rate. **(A)** Forest plot displaying the risk ratios (RRs) and 95% confidence intervals (CIs) for each study, comparing the experimental group (conventional therapy + Shoutai Pills) with the control group (conventional therapy alone). Each study is represented by a square, with its size indicating the weight of the study in the meta-analysis, and a horizontal line showing the CI. The pooled estimate, represented by a diamond, indicates a statistically significant effect favoring the experimental group (RR: 1.88, 95% CI: 1.50–2.35, p < 0.00001). No heterogeneity is observed (I^2^ = 0%, p = 0.75), demonstrating consistent findings across studies. The rightmost column provides a detailed risk of bias assessment, categorized into seven domains and visualized with green (low risk), yellow (unclear risk), and red (high risk) symbols. **(B)** Funnel plot assessing publication bias. The symmetrical distribution of data points around the central line suggests an absence of significant publication bias, further supporting the reliability of the meta-analysis results. This figure underscores the beneficial effect of the experimental intervention and highlights the methodological rigor and consistency of the included studies.

Figure 12 presents a meta-analysis of risk ratios (RRs) comparing the effectiveness of the experimental group (conventional therapy + Shoutai Pills) and the control group (conventional therapy alone). Figure 6A displays a forest plot summarizing individual study results, where each study is represented by a square (indicating the weight of the study) and a horizontal line (95% confidence interval). The pooled estimate, depicted as a diamond, shows a significant overall effect favoring the experimental group (RR: 1.88, 95% CI: 1.50–2.35, p < 0.00001). There is no evidence of heterogeneity among studies (I^2^ = 0%, p = 0.75), suggesting consistent results. The rightmost column presents the risk of bias assessment for each study, categorized into seven domains and visualized using green (low risk), yellow (unclear risk), and red (high risk) symbols. Figure 6B shows a funnel plot evaluating potential publication bias. The symmetrical distribution of studies around the central line suggests no significant publication bias, supporting the reliability of the pooled estimate. This figure highlights the significant benefit of the experimental intervention over the control and the robustness of the results due to the lack of heterogeneity and minimal publication bias (Figure 12).

#### 3.5.5 Serum D-dimer level

**FIGURE 13:**
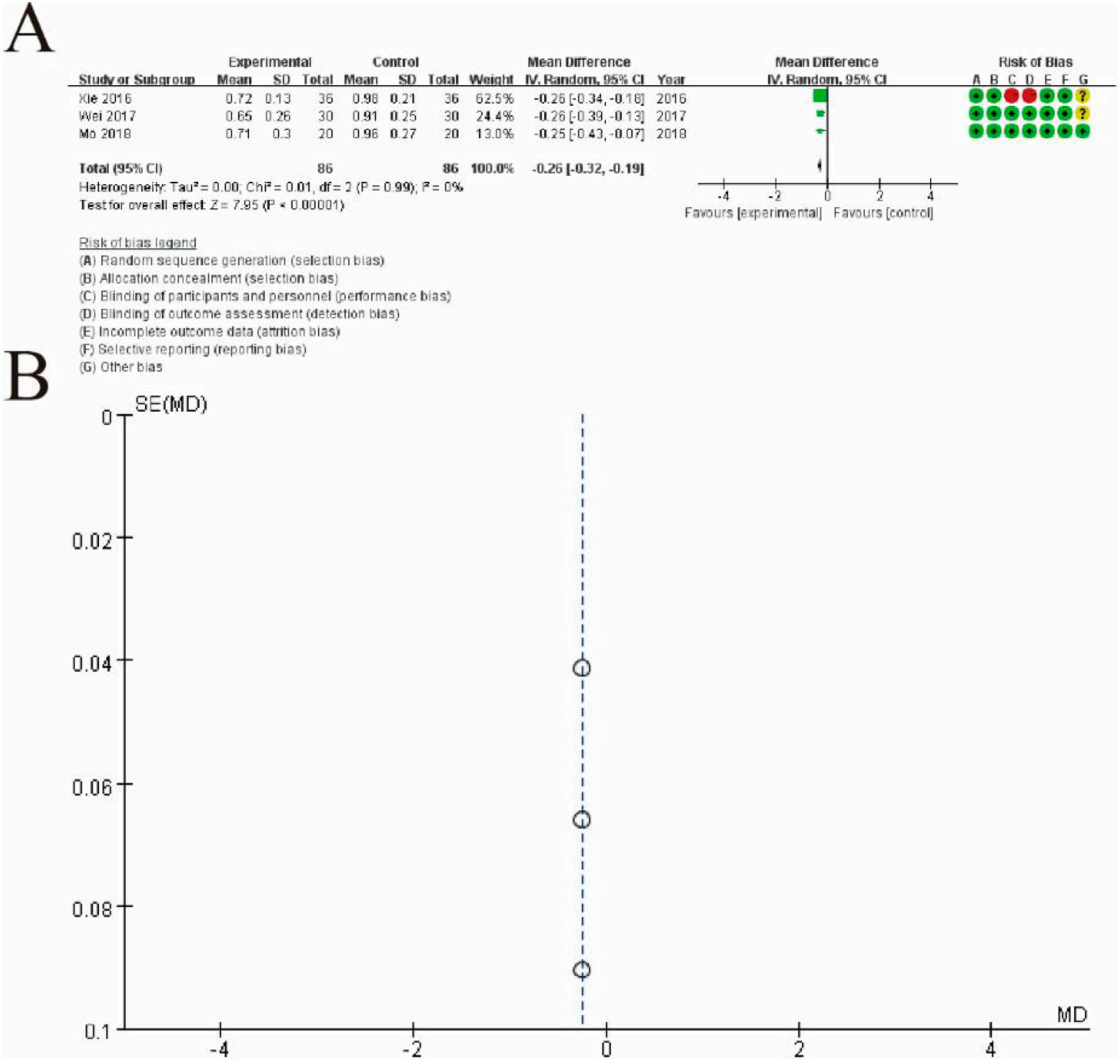
Meta-Analysis of serum D-dimer level. **(A)** Forest plot summarizing the mean differences (MDs) and 95% confidence intervals (CIs) for three included studies, comparing the experimental group (conventional therapy + Shoutai Pills) with the control group (conventional therapy alone). Each study is represented by a square, with its size reflecting the study’s weight, and a horizontal line indicating the CI. The pooled estimate, represented by a diamond, shows a statistically significant effect favoring the experimental group (MD: −0.25, 95% CI: −0.32 to −0.19, p < 0.00001). No heterogeneity is observed among the studies (I^2^ = 0%, p = 0.99), indicating consistency across findings. The risk of bias for each study is presented in the rightmost column, assessed across seven domains and visualized with green (low risk), yellow (unclear risk), and red (high risk) symbols. **(B)** Funnel plot evaluating publication bias. The symmetrical distribution of studies around the central line suggests no significant publication bias, supporting the reliability and robustness of the meta-analysis results. This figure demonstrates the significant and consistent positive effect of the experimental intervention compared to the control group.

Figure 7 presents a meta-analysis of mean differences (MDs) comparing the outcomes of the experimental group (conventional therapy + Shoutai Pills) and the control group (conventional therapy alone). Figure 7A shows a forest plot summarizing the results of three studies, where each study is represented by a square (indicating its weight in the analysis) and a horizontal line (representing the 95% confidence interval). The pooled estimate, depicted as a diamond, indicates a statistically significant overall effect favoring the experimental group (MD: -0.25, 95% CI: -0.32 to -0.19, p < 0.00001). No heterogeneity is observed among the studies (I^2^ = 0%, p = 0.99), reflecting consistency in the findings. The rightmost column provides a detailed risk of bias assessment for each study, categorized into seven domains and visualized with green (low risk), yellow (unclear risk), and red (high risk) symbols. Figure 7B displays a funnel plot assessing potential publication bias. The symmetrical distribution of the studies around the central line suggests no significant publication bias, supporting the robustness and reliability of the meta-analysis results. This figure highlights the consistent and significant benefit of the experimental intervention compared to the control group (Figure 13).

## 4 Discussion

This meta-analysis systematically evaluated the safety and clinical efficacy of Shoutai Pills in the treatment of recurrent miscarriage, synthesizing data from 11 randomized controlled trials (RCTs) involving 888 participants. The findings demonstrate that Shoutai Pills, whether used alone or in combination with conventional therapies, significantly improve pregnancy outcomes, including live birth rates and pregnancy retention rates, while exhibiting a favorable safety profile (Van den Berg et al., 2014). These results align with the principles of Traditional Chinese Medicine (TCM), which emphasize restoring bodily balance and addressing underlying deficiencies to support reproductive health (Liu et al., 2024; Wu et al., 2022). However, the limitations of the included studies, such as methodological variability and cultural specificity, highlight the need for further high-quality research to validate these findings and establish standardized protocols.

### 4.1 Clinical efficacy of Shoutai pills

The pooled analysis revealed that Shoutai Pills were associated with a nearly twofold increase in live birth rates (RR: 1.88, 95% CI: 1.50–2.35, p < 0.00001) and a significant reduction in early pregnancy loss (RR: 0.41, 95% CI: 0.33–0.51, p < 0.00001). These outcomes are clinically meaningful, as recurrent miscarriage poses significant emotional and psychological burdens on affected women. The pharmacological properties of the herbal components in Shoutai Pills, such as Dipsacus asper and Cuscuta chinensis, may contribute to these benefits. These herbs possess anti-inflammatory, antioxidant, and hormone-regulating effects, which could address immune dysregulation, oxidative stress, and hormonal imbalances—key factors implicated in recurrent miscarriage. The consistency of these findings across studies, evidenced by low heterogeneity (I^2^ = 0%), strengthens the robustness of the results.

In addition to improving pregnancy outcomes, Shoutai Pills significantly alleviated TCM syndrome scores (MD: −2.35, 95% CI: −3.32 to −1.39, p < 0.00001), which reflect symptoms related to kidney deficiency, qi stagnation, and blood imbalance. These syndromes are central to the TCM understanding of recurrent miscarriage. However, the high heterogeneity (I^2^ = 98%) observed in this outcome suggests variability in syndrome assessment and intervention protocols across studies. Future research should employ standardized diagnostic criteria and objective biomarkers to enhance the reliability of these findings.

### 4.2 Mechanistic insights

The reduction in serum D-dimer levels (MD: −0.25, 95% CI: −0.32 to −0.19, p < 0.00001) provides mechanistic support for the efficacy of Shoutai Pills. Elevated D-dimer levels are associated with hypercoagulable states, which are linked to poor pregnancy outcomes. The ability of Shoutai Pills to lower D-dimer levels suggests that they may improve uterine receptivity by regulating coagulation pathways (Shuai et al., 2024). This aligns with previous studies indicating that the anti-inflammatory and vascular-stabilizing effects of Shoutai Pills mitigate complications related to coagulation disorders. However, the limited number of studies reporting biochemical markers underscores the need for further investigation into the underlying mechanisms (Bai et al., 2023).

### 4.3 Safety profile

The safety analysis revealed no significant differences in adverse events between the Shoutai Pills and control groups, including gastrointestinal discomfort (RR: 1.04, 95% CI: 0.53–2.05, p = 0.90), fatigue (RR: 1.21, 95% CI: 0.43–3.37, p = 0.72), allergic reactions (RR: 1.20, 95% CI: 0.53–2.70, p = 0.67), and hormonal imbalance (RR: 0.60, 95% CI: 0.33–1.09, p = 0.10). The natural origin of the herbal ingredients likely contributes to their minimal side effects, making Shoutai Pills a viable complementary therapy for women who may not tolerate pharmaceutical interventions (Maharajan et al., 2021). Nevertheless, the potential for herb-drug interactions, particularly with conventional treatments like progesterone, warrants careful monitoring in clinical practice (Che et al., 2021b).

### 4.4 Limitations and future directions

Despite the promising results, this meta-analysis has several limitations. First, the included studies predominantly originated from China, where TCM is deeply integrated into clinical practice (Saab et al., 2021). This may limit the generalizability of the findings to populations with different genetic backgrounds or healthcare systems. Multicenter, international RCTs are needed to validate the efficacy and safety of Shoutai Pills in diverse populations. Second, methodological variability, such as differences in sample sizes, intervention durations, and control treatments, may introduce bias. Standardized protocols and larger sample sizes would enhance the reliability of future studies (Huang et al., 2017). Third, the subjective nature of TCM syndrome assessment complicates cross-study comparisons. Incorporating objective biomarkers and standardized tools would improve the validity of these evaluations (Zhang et al., 2015).

### 4.5 Implications for clinical practice

The integration of Shoutai Pills into modern reproductive medicine aligns with the growing trend toward holistic and patient-centered care (Wu et al., 2023). For women with unexplained recurrent miscarriage, who often have limited treatment options in conventional medicine, Shoutai Pills offer a promising complementary therapy. Their ability to improve pregnancy outcomes, alleviate symptoms, and regulate biochemical markers, coupled with a favorable safety profile, makes them a valuable addition to integrative treatment strategies. However, clinicians should exercise caution, particularly regarding herb-drug interactions, and ensure that patients use Shoutai Pills under medical supervision (Zong et al., 2014; Tang et al., 2020).

## 5 Conclusion

In conclusion, Shoutai Pills appear to offer significant benefits in improving pregnancy retention and live birth rates, alleviating TCM syndromes, and regulating biochemical markers such as D-dimer levels, all while maintaining a favorable safety profile (Qin et al., 2022). These findings provide a strong rationale for incorporating Shoutai Pills into integrative treatment strategies for recurrent miscarriage. However, further high-quality research is needed to confirm these results, elucidate underlying mechanisms, and establish standardized treatment protocols. By bridging the gap between traditional and modern medicine, Shoutai Pills have the potential to enhance outcomes for women experiencing recurrent miscarriage, offering hope and relief to a population in urgent need of effective solutions.

## Data Availability

The original contributions presented in the study are included in the article/supplementary material, further inquiries can be directed to the corresponding author.
